# Discovery of crucial cytokines associated with deep vein thrombus formation by protein array analysis

**DOI:** 10.1186/s12872-024-04030-7

**Published:** 2024-07-18

**Authors:** Qitao Wang, Junyu Chi, Wenjie Zeng, Fang Xu, Xin Li, Zhen Wang, Ming Qu

**Affiliations:** Vascular Gland Surgery, The First Affiliated Hospital of Hebei North University, Hebei province, Zhangjiakou, 075000 China

**Keywords:** Deep vein thrombus, Microarray protein, Biomarkers, Targeted therapy

## Abstract

**Background:**

Expanding the number of biomarkers is imperative for studying the etiology and improving venous thromboembolism prediction. In this study, we aimed to identify promising biomarkers or targeted therapies to improve the detection accuracy of early-stage deep vein thrombosis (DVT) or reduce complications.

**Methods:**

Quantibody Human Cytokine Antibody Array 440 (QAH-CAA-440) was used to screen novel serum-based biomarkers for DVT/non-lower extremity DVT (NDVT). Differentially expressed proteins in DVT were analyzed using bioinformatics methods and validated using a customized array. Diagnostic accuracy was calculated using receiver operating characteristics, and machine learning was applied to establish a biomarker model for evaluating the identified targets. Twelve targets were selected for validation.

**Results:**

Cytokine profiling was conducted using a QAH-CAA-440 (RayBiotech, USA) quantimeter array. Cross-tabulation analysis with Venn diagrams identified common differential factors, leading to the selection of 12 cytokines for validation based on their clinical significance. These 12 biomarkers were consistent with the results of previous array analysis: FGF-6 (AUC = 0.956), Galectin-3 (AUC = 0.942), EDA-A2 (AUC = 0.933), CHI3L1 (AUC = 0.911), IL-1 F9 (AUC = 0.898), Dkk-4 (AUC = 0.88), IG-H3 (AUC = 0.876), IGFBP (AUC = 0.858), Gas-1 (AUC = 0.858), Layilin (AUC = 0.849), ULBP-2 (AUC = 0.813)and FGF-9 (AUC = 0.773). These cytokines are expected to serve as biomarkers, targets, or therapeutic targets to differentiate DVT from NDVT.

**Conclusions:**

EDA-A2, FGF-6, Dkk-4, IL-1 F9, Galentin-3, Layilin, Big-h3, CHI3L1, ULBP-2, Gas-1, IGFBP-5, and FGF-9 are promising targets for DVT diagnosis and treatment.

**Supplementary Information:**

The online version contains supplementary material available at 10.1186/s12872-024-04030-7.

## Background

Venous thromboembolism (VTE), encompassing both deep vein thrombosis (DVT) and pulmonary embolism (PE), most commonly occurs in the lower limbs, accounting for up to 50% of cases [1] DVT affects approximately 10 million people worldwide annually [2]. Several risk factors, such as trauma, atherosclerosis, and cancer, can influence DVT development. Presently, DVT is primarily diagnosed using Doppler ultrasonography (DU) and D-dimer [2]. However, the concentration of D-dimer increases with age, decreasing its specificity for venous thromboembolism diagnosis [3]. DU remains the primary diagnostic tool for DVT [4].

Currently, there are no accurate laboratory indicators and detection methods for early-stage DVT and its prediction in clinical settings. DVT remains a leading cause of postoperative morbidity and mortality in patients who have sustained injuries or undergone surgery. Despite the use of anticoagulation therapy according to guidelines, most injured patients, such as those with fractures, still develop DVT [5]. This not only results in significant economic costs but also leads to severe complications such as post-thrombotic syndrome (PTS) [6] and intracranial bleeding [7]. The incidence of PTS can be as high as 50%, with 5% of cases progressing to severe PTS [6], consequently reducing quality of life and incurring substantial healthcare costs.

Currently, DVT treatment includes anticoagulant and catheter-directed therapies, both of which carry an increased risk of hemorrhage, as well as advantages and disadvantages [8, 9]. Therefore, prevention and early-stage diagnosis should be considered our long-term goal. Expanding the number of biomarkers is imperative for studying the etiology and improving VTE prediction. We employed Quantibody^®^ Human Cytokine Antibody Array 440 (Catalog #: QAH-CAA‐440, RayBiotech Inc) to compare serum protein in patients with DVT and without DVT after fracture under standard coagulation conditions. This study aims to identify new biomarkers for the prediction or diagnosis of early-stage DVT to decrease the incidence of DVT complications. We hope that this method will aid in discovering novel mechanisms and therapeutic targets for the study of DVT.

## Methods

### Subjects and samples

Patient specimens were collected with approval from the Institutional Ethics Committee of the First Affiliated Hospital of Hebei Northern University. Informed consent was obtained according to the Declaration of Helsinki. All patient samples were de-identified and re-labeled to maintain patient privacy. This study was approved by the First Affiliated Hospital of Hebei North University (approval number: R2020240). All patients were appropriately anticoagulated with heparin according to the guidelines. Serum samples were collected within 3 d of disease onset. Three groups were matched by age, sex, and complications. Diagnostic methods for DVT included the D-dimer test, ultrasound, and imaging. Common risk factors such as cancer, immune diseases, oral estrogen, and pregnancy were excluded [2]. These patients had no severe conditions such as abnormal liver or kidney function or cardiocerebrovascular disease.

After overnight fasting, blood samples were collected from each participant within 3 d of their acute thrombosis diagnosis into sodium citrate collection tubes. The serum was separated and stored at − 80℃ until extraction.

### Protein antibody array and custom array for 12 targets

Protein lysates from DVT serum samples (100 µL) were quantitatively analyzed using antibody arrays for 440 cytokines, cytokine receptors, and related proteins (details in Supplementary Table S1, GSM-CAA-440, RayBiotech, USA) according to the manufacturer’s instructions. The protein array slides were incubated with the protein lysates, biotin-labeled antibodies, and Cy3-conjugated streptavidin. The plates were scanned with a microarray scanner (InnoScan 300, Innopsys, France) at the Cy3 wavelength, and fluorescence intensity data were analyzed using the Q-Analyzer Tool specific to this array. Differentially expressed proteins (DEPs) were then identified. Twelve proteins identified by QAH-CAA‐440 were selected for evaluation using a custom antibody array (CA20: Human Custom Antibody, RayBiotech Inc.) with 36 subjects (15 DVT, 15 non-lower extremity DVT [NDVT], and 6 normal controls [NC], including the 25 subjects from the biomarker discovery stage). The serum levels of these 12 proteins were measured according to the manufacturer’s instructions.

### ELISA

ELISA kits (ABclonal, Wuhan, CN) were used to validate the antibody array results following the manufacturer’s instructions. Briefly, serum samples were diluted at varying dilution factors based on individual serum biomarkers. The diluted samples were then coated onto the plates and incubated overnight at 4 °C. The plates were washed with wash buffer, and a biotin-conjugated antibody (1:100) was added to the ELISA plate and incubated for 2 h. Subsequently, horseradish peroxidase–conjugated streptavidin (1:100) was added to catalyze the TMB reagent. The catalytic reaction was stopped by adding a stop solution. Each step involved incubating 100 µL per well. Finally, OD_450_ was measured using a microplate reader (RT-6100, Rayto, Shenzhen, CN).

### Function enrichment analysis

The identified DEPs in DVT were analyzed using DAVID to determine enriched terms related to Gene Ontology (GO) and the Kyoto Encyclopedia of Genes and Genomes (KEGG) [10]. Briefly, GO annotations were conducted to reveal the biological attributes of the DEPs, while the KEGG database was consulted to identify the significant pathways involving the DEPs using clustering algorithms.

### Bioinformatic analysis

All array data were analyzed using RayBiotech Analysis Tool software (Q-Analyzer Software for QAH‐CAA‐440) (https://www.raybiotech.com/products/other‐products/software/). We conducted GO and pathway analyses to elucidate the potential functions of the DEPs identified from the Quantibody Array. These analyses described the biological processes, cellular components, and molecular functions by inputting the gene IDs of the DEPs into the KOBAS 3.0 database (http://kobas.cbi.pku.edu.cn/index.php). Additionally, protein–protein interaction (PPI) analysis was conducted using the STRING database (https://string-db.org/cgi/input.pl), with protein IDs used to identify node proteins.

### Statistical analysis

Statistical analysis was conducted using SPSS v. 24.0. Results are presented as means ± SD. Results with a fold change (FC) > 1.2 or < 0.83, *t*-test *p*-value < 0.05, and a fluorescence value > 150 between the two groups were considered significantly different.

## Results

### General features and identification of DEPs

A case-control study was conducted on patients with DVT (*n* = 10), NDVT (*n* = 10), and NCs (*n* = 5). The participants’ demographic data, including age, sex, body mass index (BMI), and medical history, are presented in Table 1. Significant differences in sex, BMI, hypertension, dyslipidemia, and stroke were observed between the DVT and NDVT groups. All participants underwent cytokine profile screening using the Quantibody Array QAH-CAA‐440 (RayBiotech, USA), which quantitatively measured the expression levels of 440 cytokines in these serum samples. DEPs were defined as having a signal intensity FC > 1.2 (upregulated) or < 0.83 (downregulated).

**Fig. 1:**
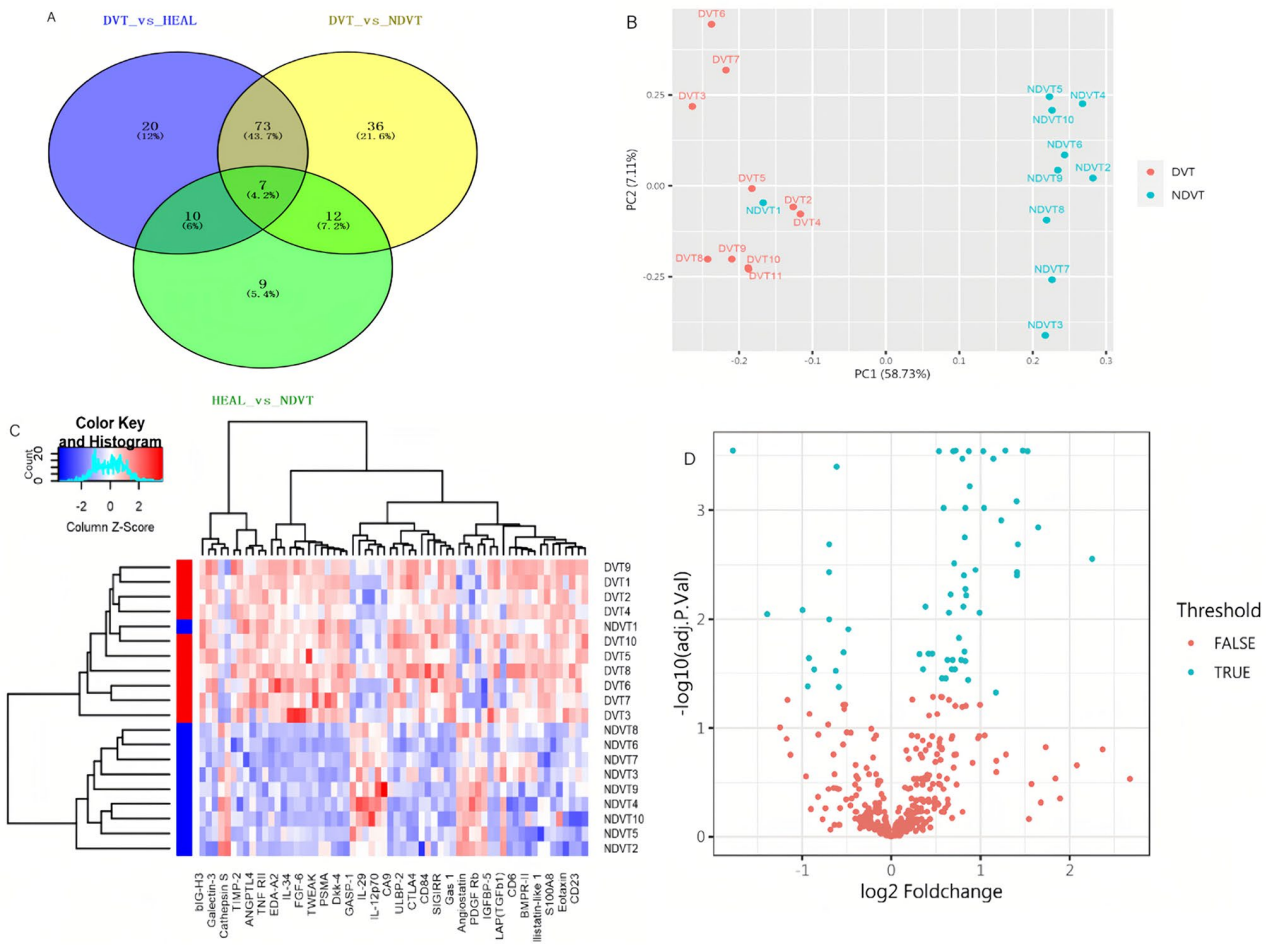
DVT-specific biomarker analysis **(A)** Venn diagram analysis. The proteins differentially expressed among the DVT, NDVT, and control groups were analyzed using a Venn diagram to identify DVT-specific biomarkers. The blue circle represents DVT versus control, the yellow circle represents DVT versus NDVT, and the green circle represents NDVT versus control. **(B)** Principal component analysis of DVT and NDVT. The DVT samples (red) are sharply differentiated from the NDVT samples (blue) on the second principal component. **(C)** Hierarchical clustering heatmap of proteins in DVT and NDVT. Protein levels are depicted as colors ranging from blue (low concentration) to white (intermediate concentration) to red (high concentration), according to the mean of each protein. The group of samples is shown on the right and by the colored bar on the left (red represents DVT, and blue represents NDVT). **(D)** Volcano plot of candidate protein expression in DVT and NDVT from the Human Antibody Array QAH-CAA-440. Proteins are visualized according to their log FC (*x*-axis, FC) and significance (*y*-axis: −log10 adj. *p*. val) in the DVT and NDVT groups. Proteins highlighted in blue had adj. *p*. val < 0.05 and fold change > 1.2 or < 0.83 in the dataset

To identify specific biomarkers for DVT, DEPs were statistically analyzed using one-way analysis of variance, followed by multiple comparisons with a post hoc Bonferroni test between any pair of the DVT, NDVT, and NC groups. Consequently, 62 proteins were found to be differentially expressed between the DVT and NDVT groups (detailed in Supplementary Table S1), 59 between the DVT and NC groups (Table S2), and 2 between the NDVT and NC groups (Table S3). A Venn diagram (Fig. 1A) identified 36 specific DVT-associated biomarkers.

Differentiated proteins were statistically analyzed using multifactor, multigroup crossover analysis to re-identify the selected DVT-specific biomarkers, followed by multiple false discovery rate comparisons among the DVT, NDVT, and NC groups results confirmed that the 12 chosen DEPs were still included in the filtered datasets (Table S4).

**Table 1:** Baseline characteristics of participants in the three groups

	DVT (*n* = 10)	NDVT (*n* = 10)	Control (*n* = 5)	DVT-NDVT *p*-value	DVT-Control *p*-value	NDVT-Control *p*-value
Age, mean ± SD	60.50 ± 11.11	60.40 ± 13.92	58.20 ± 4.60	0.98	0.72	0.73
Male, *n* (%)	14.90%	20.90%	7.90%	0.65	0.27	0.61
BMI, mean ± SD	23.55 ± 2.98	24.71 ± 3.67	23.65 ± 2.62	*p* < 0.001	0.66	*p* < 0.001
Smoking, *n* (%)	20%	20%	20%	0.71	1.00	0.71
Hypertension, *n* (%)	55.9%	21.5%	59.5%	*p* < 0.001	0.31	*p* < 0.001
Dyslipidemia, *n* (%)	0.00%	12.4%	21.3%	*p* < 0.001	*p* < 0.001	0.001
Diabetic, *n* (%)	24.10%	19.90%	0.00%	0.08	*p* < 0.001	*p* < 0.001
Stroking, *n* (%)	10.60%	22.8%	0.00%	*p* < 0.001	*p* < 0.001	*p* < 0.001
Coronary heart disease, *n* (%)	12.1%	12.4%	0.00%	0.861	*p* < 0.001	*p* < 0.001

DEPs were defined as those with an adjusted *p*-value (adj. *p*. val) < 0.05 and FC > 1.2 or < 0.83 (absolute log fc > 0.263)

Principal component analysis was conducted on all DEPs to compare DVT-specific components with those of the NDVT and NC groups (Fig. 1B).

Our results show significant differences in cytokine expression among the three groups, with acceptable consistency within each group. The volcano plot highlights 62 DEPs based on an adj. *p*. val < 0.05. Of these, 48 proteins were upregulated, and 14 were downregulated (Fig. 1C). To determine if DEPs could distinguish between the DVT, NDVT, and NC groups, we conducted a heatmap of hierarchical clustering analysis. The results indicated that most DVT samples could be distinguished from the NDVT group to form two major clusters, and that the two clusters were isolated based on the differential expression of proteins (Fig. 1C).

### Bioinformatics analysis

**Fig. 2:**
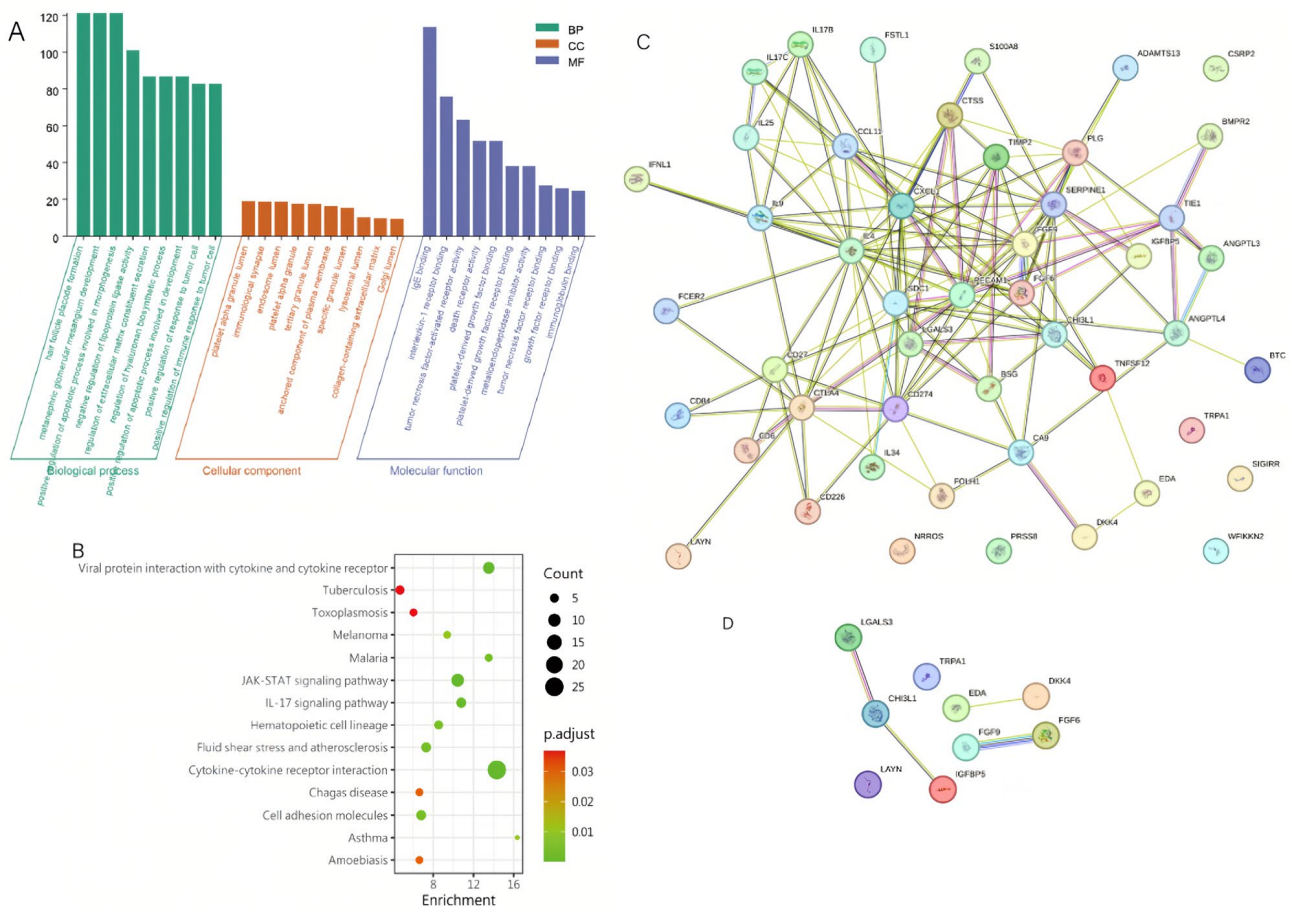
**(A)** GO terms are related to biological processes. **(B)** KEGG pathway analysis indicates the significant pathways enriched by DEPs between two groups. The *x-*axis shows the amounts of proteins associated with the KEGG pathway, with colors ranging from green to red, representing different levels of adj. *p*-values. **(C)** and **(D)** PPI network analysis was conducted for the 62 DEPs **(C)** and 36 specific proteins **(D)**. Lines between two proteins indicate correlations in biological function, with line thickness reflecting the strength of data support

DEPs were used for GO and KEGG enrichment analyses. Protein function enrichment was analyzed using the R package “clusterProfiler.” The 10 DEPs with the highest credibility were filtered according to their *p*-values and included in a three-in-one GO map. GO biological process analysis revealed that the DEPs (DVT vs. NDVT) were involved in responses to molecules of bacterial origin, response to lipopolysaccharide, positive regulation of cytokine production, peptidyl-tyrosine modification, neutrophil migration, neutrophil chemotaxis, leukocyte migration, and leukocyte chemotaxis (Fig. 2A). Notably, numerous cytokines were related to inflammation in both groups.

**Fig. 3:**
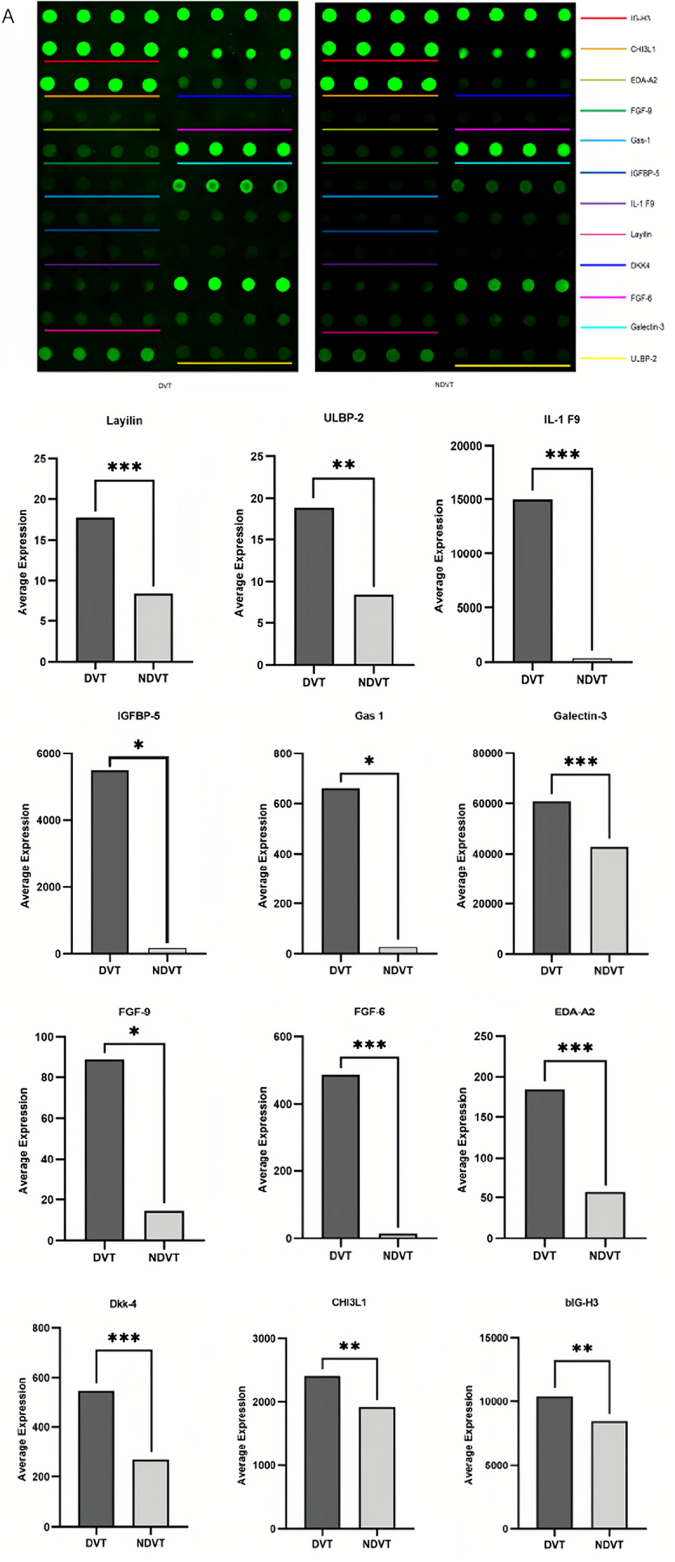
**(A)** Validation of serum proteins from patients with DVT using the customized array. **(A)** The fluorescence intensity profiles from the customized arrays depict protein levels proportional to their fluorescence. Each antibody was printed in four duplicates, and serum protein locations for the NC, DVT, and NDVT groups are highlighted in colored boxes. **(B)** Histogram of customized array data showing 12 DEFs across the DVT, NDVT, and NC groups. *p < 0.05 compared to NDVT or NC groups

**Fig. 4:**
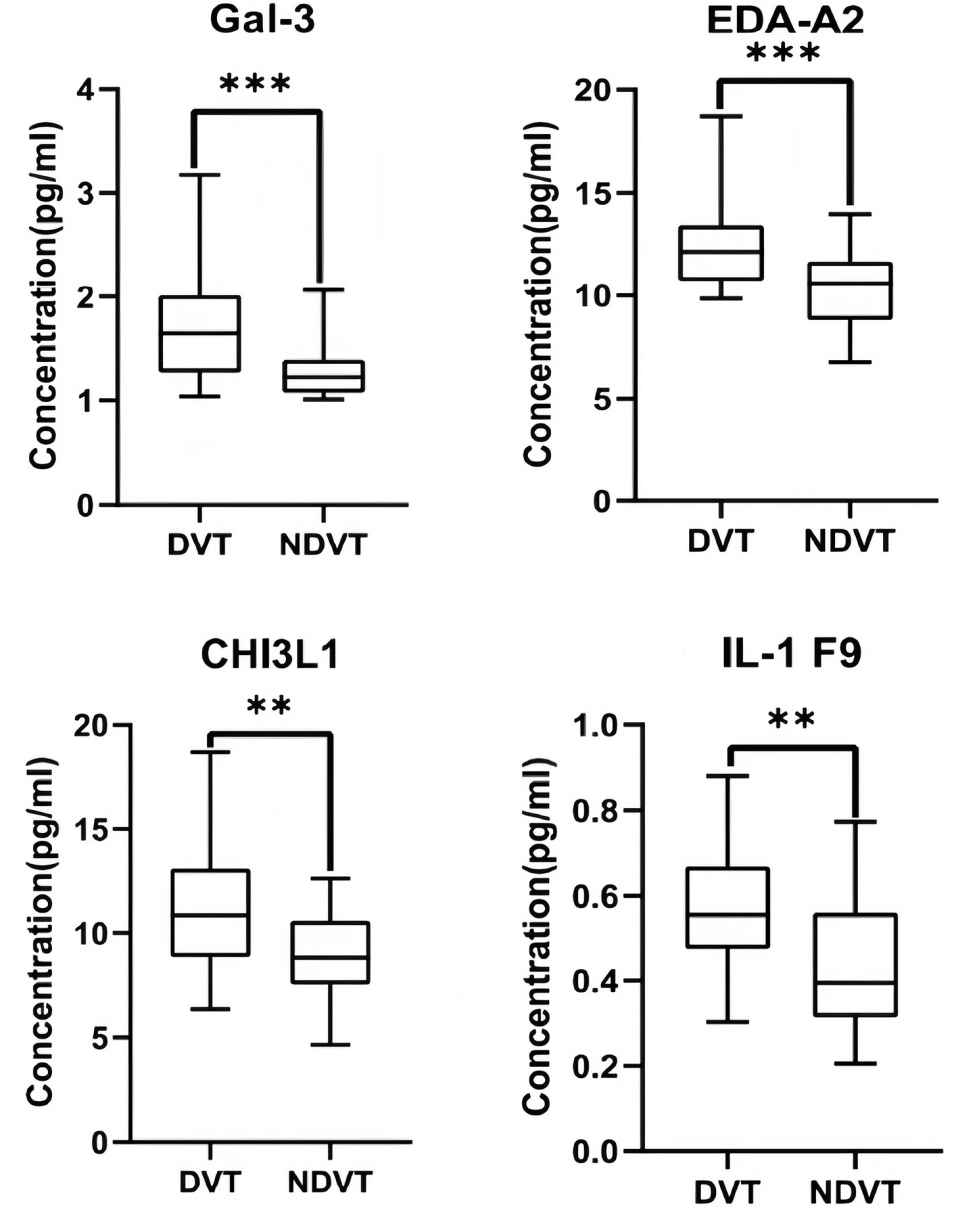
ELISA validation results of serum DVT biomarkers were illustrated using Boxplot. The centerline in the boxplot represents the median of data within each group. The *p*-values comparing DVT versus NDVT for each protein were obtained using Mann–Whitney *U* test analysis. ***p < 0.0001, **p < 0.001, and *p < 0.05

We conducted a KEGG pathway analysis to gain further insight into the pathways involving DPEs. Fourteen KEGG pathways showed statistical significance (*p* < 0.05) (Fig. 2B). The biologically regulated pathways with significantly different changes in DVT versus NDVT involved viral protein interactions with cytokines and cytokine receptors, tuberculosis, toxoplasmosis, melanoma, malaria, JAK-STAT signaling pathway, the IL-17 signaling pathway, among others (Fig. 2B). Interactions between DEPs are illustrated in Fig. 4C and D.

### Verification of essential proteins

Intersection analysis using Venn diagrams revealed common differential factors. Based on their clinical significance, 12 cytokines associated with DVT were selected compared to the NDVT and NC groups (customized array criteria: *p* < 0.05, FC > 0.83). However, these proteins have not been previously studied in patients with DVT. We recruited 10 DVT, 10 NDVT, and 5 NC cases to validate these 12 proteins. The expression levels of EDA-A2, FGF-6, Dkk-4, IL-1 F9, Galectin-3, Layilin, bIG-H3, CHI3L1, ULBP-2, Gas-1, IGFBG-5, and FGF-9 were upregulated in the DVT group, consistent with the initial screening results. Quantitative fluorescence intensity data were obtained from scanned images of the array slides (Fig. 3A, B).

### Validation of array results using ELISA

Additional samples, including 30 NC cases and 30 DVT samples, were analyzed using ELISA to validate the specific biomarkers associated with DVT. Among the 12 DVT-related biomarkers, (EDA-A2, CHI3L1, Gal-3, and IL-1 F9) were selected for ELISA verification due to their limited sample size. The levels of these cytokines measured using ELISA were consistent with the array results, confirming differential expression between the DVT and NC groups (Fig. 4).

### Sensitive and specific analysis of several biomarkers

**Fig. 5:**
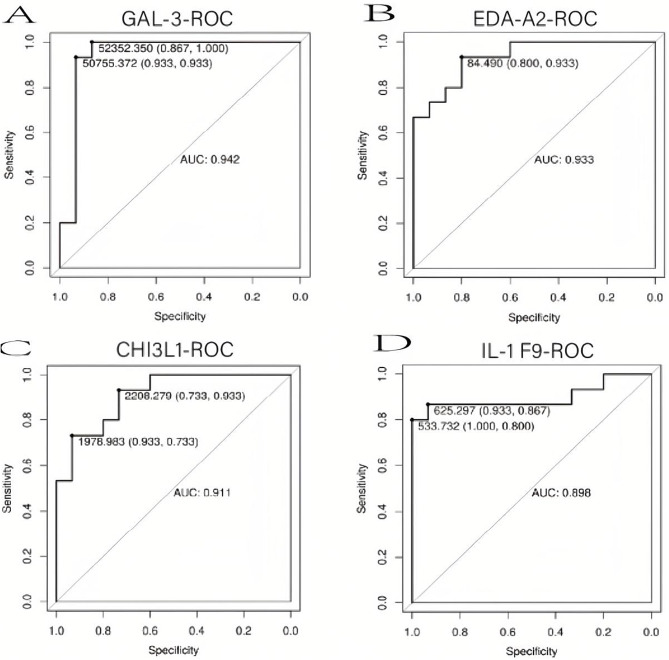
ROC curve analysis showed that the four serum cytokines were differentially expressed in patients with DVT. The ROC curve summarizes the accuracy of cytokines in predicting DVT presence, with the AUC indicating the average sensitivity of the biomarker. An AUC of 0.5 suggests no predictive value for the biomarkers, while an AUC of 1 implies the biomarkers’ perfect predictive ability. **(A)** AUC of Gal-3 (0.942). **(B)** AUC of EDA-A2 (0.993). **(C)** AUC of CHI3L1 (0.911). **(D)** AUC of IL-1 F9 (0.898)

ROC curves were used to assess the sensitivity and specificity of 12 proteins in differentiating between the DVT and NDVT groups. The proteins and their corresponding AUC are as follows: FGF-6 (0.956), Galectin-3 (0.942), EDA-A2 (0.933), CHI3L1 (0.911), IL-1 F9 (0.898), Dkk-4 (0.88), IG-H3 (0.876), IGFBP (0.858), Gas-1 (0.858), Layilin (0.849), ULBP-2 (0.813), and FGF-9 (0.773). Combined with clinical significance, Galectin-3, EDA-A2, CHI3L1, and IL-1 F9 had the highest sensitivity, specificity, and AUC values, indicating their superior diagnostic performance in differentiating between patients with DVT and patients with NDVT (Fig. 5).

## Discussion

VT results from the interaction of various factors, such as vascular endothelial cells [11], platelets [12], coagulation function [13], and the fibrinolytic system [14], all of which undergo varying degrees of change before thrombosis [15]. Therefore, it is crucial to implement appropriate interventions to hinder VT development and to identify early screening and diagnostic methods. Twelve target proteins were identified for further validation. In the DVT group, the expression levels of EDA-A2, FGF-6, Dkk-4, IL-1 F9, Galectin-3, Layilin, bIG-H3, CHI3L1, ULBP-2, Gas-1, IGFBG-5, and FGF-9 were upregulated, consistent with the initial screening results. These differential proteins could serve as specific markers for DVT, facilitating early diagnosis and treatment, thereby assisting clinicians in formulating more effective evaluation and treatment strategies.

### Chitinase-3-like protein 1

Chitinase-3-like protein 1 (CHI3L1), a 39 kDa slip-membrane protein, is an inflammatory glycoprotein in various human cancers. Elevated serum CHI3L1 levels are biomarkers of poor prognosis in patients with advanced cancers. Its overexpression in SW480 (human colon carcinoma cells) enhances THP1 cells (human macrophages) and migration of human umbilical vein endothelial cells (HUVECs), as well as tube formation of HUVECs [16]. CHI3L1 expression is elevated in various inflammatory and chronic diseases, including obesity, diabetes, kidney disease, rheumatoid arthritis, inflammatory bowel disease, cancer, coronary artery disease, and acute ischemic stroke [16–21]. CHI3L1 plays a crucial role in vascular inflammation and atherosclerosis development by promoting EC inflammation and vascular smooth muscle cells migration and value-addition [22].

### Galectin-3

Galectin-3, a 60-90-kDa protein, is distributed in the epithelial and myeloid/amoeboid cells [23]. Galectin-3 and its receptor are secreted proteins that promote inflammatory cascades among inflammatory pathways [24]. In a prospective study, the plasma galectin-3 concentration was positively correlated with the incidence of VTE after adjusting for risk factors [25]. DeRoo et al. identified the significant role of this protein in VTE, correlating thrombosis size with gal3 concentration. Galectin-3-binding protein levels rapidly increase during acute VTE. In this study, animal models of VTE showed that gal3 and gal3bp were broadly distributed in vein walls, red blood cells, platelets, and microparticles, with gal3 present in leukocytes [24]. Although this evidence proves that gal3 is positively associated with DVT, this remains to be validated.

### EDA-A2

EDA-A2, a member of the tumor necrosis factor (TNF) ligand family, is a ligand for the X-linked ectodermal dysplasia receptor (EDA2R) [26, 27], which has been reported to activate classical nuclear factor-κappa B (NF-κB) signaling and mitogen-activated protein (MAP) kinases. EDA2R belongs to the TNF receptor family, which is involved in various signaling pathways, including immune response, inflammation, development, and carcinogenesis [28]. It has been demonstrated that EDA-A2 promotes apoptotic signaling by binding to the EDA2R receptor. However, the role of EDA-A2 in DVT has not been investigated.

### IL1-F9

IL-1 is a pleiotropic cytokine that initiates immune and inflammatory responses in nearly all body tissues [29–32]. It activates transcriptomic factors, like NF-κB and AP-1, as well as MAPKs such as JNK and p38, leading to the production of various cytokines, chemokines, adhesion molecules, and enzymes such as cyclooxygenase and carbon monoxide synthase [33, 34]. IL1-F9 has recently been shown to activate NF- κB in an IL-1Rep2-dependent manner and signals similarly to IL-1, inducing many of the same downstream effects. However, the role of IL1-F9 in DVT has not yet been clarified experimentally [35].

## Conclusion

In conclusion, EDA-A2, FGF-6, Dkk-4, IL-1 F9, Galectin-3, Layilin, bIG-H3, CHI3L1, ULBP-2, Gas-1, IGFBG-5, and FGF-9 were differentially expressed in patients with DVT compared to those in the NDVT and NC groups. This study has some limitations due to its relatively small sample size. Multicenter cohorts with larger sample sizes of DVT, NDVT, and NC are needed to evaluate the diagnostic potential of these 12 biomarkers in the prethrombotic state following a fracture. Further studies are required to explore the mechanisms linking postfracture and prethrombotic states. Combining protein array technology with traditional laboratory indicators can improve the accuracy, specificity, and sensitivity of diagnosing postfracture thrombosis.

### Electronic supplementary material

Below is the link to the electronic supplementary material.

Supplementary Material 1

Supplementary Material 2

Supplementary Material 3

Supplementary Material 4

## Data Availability

No datasets were generated or analysed during the current study.

## Abbreviations

AUC: Area Under the Curve
BMI: Body Mass Index
CHI3L1: Chitinase-3-like protein 1
DVT: Deep Vein Thrombosis
DU: Doppler Ultrasound
EC: Endothelial Cell
EDA: A2-Ectodysplasin A2
ELISA: Enzyme-Linked Immunosorbent Assay
FGF: 6-Fibroblast Growth Factor 6
FGF: 9-Fibroblast Growth Factor 9
FDR: False Discovery Rate
GAS: 1-Growth Arrest-Specific 1
GO: Gene Ontology
IG: H3-Immunoglobulin-H3
IGFBP: 5-Insulin-like Growth Factor Binding Protein 5
IL: 1 F9-Interleukin-1 Family Member 9
KEGG: Kyoto Encyclopedia of Genes and Genomes
NDVT: Non-Deep Vein Thrombosis
PC2: Principal Component 2
PCA: Principal Component Analysis
PE: Pulmonary Embolism
PPI: Protein-Protein Interaction
PTS: Postthrombotic Syndrome
ROC: Receiver Operating Characteristics
SD: Standard Deviation
SPSS: Statistical Package for the Social Sciences
STRING: Search Tool for the Retrieval of Interacting Genes/Proteins
ULBP: 2-UL16 Binding Protein 2
VTE: Venous Thromboembolism
VEGF: Vascular Endothelial Growth Factor
